# Experimental Study on Thermal Oxidative Aging Effects on the Performance and Compatibility of Different Types of Waterproofing Membranes

**DOI:** 10.3390/polym18020162

**Published:** 2026-01-07

**Authors:** Shaochun Li, Yang Du, Wenbin Geng, Ruiyun Zhang, Guojun Sun, Xingpeng Ma

**Affiliations:** 1 China Electronics Engineering Design Institute Co., Ltd., Beijing 100142, China; lishaochun@sdic.com.cn; 2 China Electricity Council, Beijing 100761, China; 3 China Construction Sixth Engineering Bureau Co., Ltd., Tianjin 300012, China; 4 Faculty of Architecture, Civil and Transportation Engineering, Beijing University of Technology, Beijing 100124, China

**Keywords:** waterproofing membrane, thermal-oxidative aging, compatibility, low-temperature flexibility, mechanical property

## Abstract

As urbanization and extreme weather conditions intensify, the comprehensive performance requirements for building waterproofing systems are becoming more demanding. Single-layer waterproof membranes often struggle to meet usage requirements in complex environments, leading to the gradual rise of composite waterproof systems. This paper selects three different types of waterproof membranes, ultra-thin reinforced self-adhesive polymer-modified bitumen waterproof membrane, polymer self-adhesive waterproof membrane, and polymer-modified bitumen root penetration-resistant waterproof membrane, and conducts a systematic study on their compatibility and durability. Through tensile performance, low-temperature flexibility, and peel compatibility tests, combined with thermal oxidative aging experiments at different aging times, the mechanical behavior, low-temperature adaptability, and interfacial bonding characteristics of the membranes were analyzed. The results show that the three membranes differ significantly in tensile performance. The root penetration-resistant membrane has the highest strength but is more brittle, the polymer self-adhesive membrane has lower strength but better stability, and the ultra-thin reinforced membrane performs better initially but lacks durability. In terms of low-temperature flexibility, the root penetration-resistant membrane demonstrates superior crack resistance and aging resistance. These divergent aging responses are closely related to differences in reinforcement structure, polymer modification, and the thermal–oxidative sensitivity of the bituminous adhesive layers. Peel compatibility tests show that the peel strength of the composite membranes of the ultra-thin reinforced and polymer self-adhesive membranes is significantly improved, indicating a good synergistic effect and compatibility. Overall, different waterproof membranes exhibit distinct compatibility mechanisms and aging patterns in composite applications, providing a scientific basis for the design and optimization of composite waterproof systems.

## 1. Introduction

With the rapid acceleration of urbanization and the growing frequency of extreme weather events, waterproofing systems have become increasingly critical in construction, water conservancy, and underground engineering. In particular, in applications such as underground structures, rooftops, and basements [1,2], waterproof membranes serve as the core components of waterproofing systems, effectively preventing moisture penetration and structural deterioration. In addition to blocking water infiltration, these membranes also provide resistance to chemical corrosion, temperature fluctuations, and external mechanical stresses [3]. However, in modern buildings and infrastructure systems, the waterproofing performance of single materials is often insufficient to meet the demands of complex service environments, where different structural components and external conditions impose diverse performance requirements.

**Figure 1:**
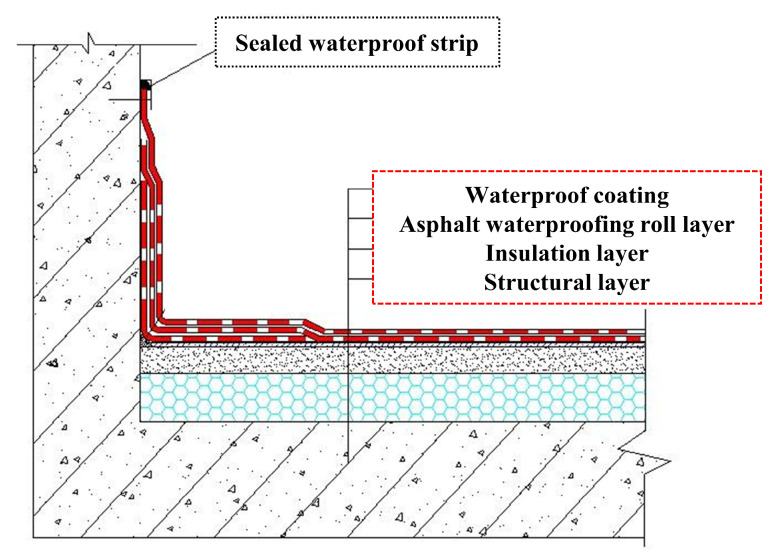
Schematic diagram of the composite waterproofing approach.

Consequently, the combined use of multiple waterproof materials has become a widely adopted strategy in building waterproofing, as it allows different layers to function synergistically and significantly enhances overall system performance. In this context, compatibility refers to the ability of combined materials to form a stable and integrated system without interfacial repulsion or delamination. In practical engineering applications, compatibility is primarily reflected in the coordination and matching of different materials within the waterproofing system and plays a critical role throughout all stages of construction and service. First, the substrate and interface treatment agents must be compatible with the primary waterproof layers to ensure the formation of a reliable bonding interface; otherwise, adhesion strength and overall waterproofing performance may be significantly compromised. Second, at critical weak points such as edges and joints where membranes intersect with coatings, sealing materials must also exhibit high compatibility with the primary waterproof materials to effectively mitigate leakage risks arising from stress concentration and interfacial failure. Furthermore, during the service life of the waterproofing system, repair strategies adopted in response to damage or degradation also depend strongly on material compatibility. Ideally, materials with properties identical or highly similar to those of the original waterproof layer should be prioritized to maintain system consistency to the greatest extent possible. However, when repair space is limited or construction conditions do not permit such matching, the use of alternative materials must still adhere to compatibility principles to ensure stable bonding with the original system and maintain the overall protective performance of the waterproofing system [4], as illustrated in Figure 1.

Since the implementation of the mandatory provisions of the General Code for Waterproofing of Building and Municipal Engineering (GB 55030-2022) [5], waterproof performance requirements have become increasingly stringent, and greater attention has been paid to the compatibility of composite waterproofing systems. Consequently, both domestic and international researchers have conducted extensive studies on the compatibility of waterproof membranes [6]. Li et al. [7] investigated the compatibility between rejuvenators and aged bitumen and proposed a solubility evaluation method based on electrical conductivity. This method effectively characterizes the interactions between rejuvenators and aged bitumen, thereby providing a valuable reference for optimizing asphalt rejuvenation technologies. To address the aging behavior of SBS-modified bitumen under high-temperature and high-humidity conditions, Liu et al. [8] conducted thermal oxidative aging and wet–dry cycling experiments. Using multiple performance indicators, including permeability, softening point ratio, and ductility, they analyzed the aging evolution mechanisms and elucidated the influence of additive content on the aging behavior of modified bitumen. Furthermore, Ren et al. [9] employed fluorescence microscopy and Fourier-transform infrared spectroscopy to investigate the short-term aging mechanisms of SBS-modified bio-asphalt. Their results indicated that early-stage aging was primarily attributed to the volatilization of light components and the thermal oxidative degradation of SBS. Although bio-asphalt can slow the aging process, it does not alter the fundamental aging mechanism.

Beyond macroscopic performance deterioration, the durability degradation of bituminous coating systems is fundamentally governed by underlying chemical degradation mechanisms. Recent studies indicate that thermo-oxidative aging of bitumen predominantly follows a free-radical chain reaction mechanism, wherein oxygen and thermal or photo energy initiate radical formation, leading to chain scission, molecular agglomeration, and the generation of carbonyl and sulfoxide functional groups [10]. Meanwhile, exposure to humid environments introduces additional degradation pathways associated with moisture-induced and hydrolytic processes. Moisture can diffuse into the bitumen matrix, interact with polar functional groups, and form bonded or clustered water states, thereby disrupting the colloidal structure of bitumen and accelerating aging. Importantly, moisture and oxygen do not act independently; rather, their transport and reaction processes are strongly coupled, as oxidative aging increases material polarity and consequently enhances moisture uptake, further intensifying degradation [11].

In research on composite materials for waterproofing systems, the application of novel waterproof coatings has attracted increasing attention. Xiao et al. [12] developed acrylic waterproof coatings and demonstrated that the resulting membranes effectively inhibit the migration of low-molecular-weight oils in bitumen, thereby extending the service life of waterproofing systems. Zhang et al. [13] investigated the bonding and peel performance of composite waterproof membranes and coatings, providing preliminary insights into their compatibility and offering guidance for waterproofing system design. The Technical Code for Roof Engineering further stipulates that waterproof membranes and coatings selected for composite waterproof layers must exhibit good compatibility [14]. Lan et al. [15] reported that polyurethane coatings exhibited poor peel performance when combined with various waterproof membranes, particularly in the absence of sufficient experimental validation, and therefore advised against their direct composite application. However, by employing specialized construction techniques to combine polyurethane coatings with self-adhesive TPO membranes, effective root resistance was achieved, demonstrating the potential advantages of this combination in specific applications [16]. Li et al. [17] further investigated the peel performance of polyurethane coatings combined with self-adhesive polymer-modified bitumen membranes under different curing times, oil filler types, and prepolymer contents. Their results indicated that increasing prepolymer content promoted the formation of a more ordered three-dimensional network structure, resulting in a denser coating surface and significantly enhanced mechanical properties, with peel strength exceeding 1.0 N/m.

Micro-scale degradation processes, such as polymer chain scission and oxidation, directly influence the macroscopic compatibility behavior of SBS-modified bitumen. These degradation mechanisms affect both mechanical properties and interfacial bonding, thereby establishing a direct link between molecular aging and the overall performance of composite systems. This relationship reinforces the theoretical continuity between previous studies on bitumen aging and the present investigation into membrane compatibility.

Regarding the performance optimization of modified bitumen, SBS-modified bitumen incorporating active rubber powder exhibits excellent high-temperature rheological properties and aging resistance while maintaining a stable multiphase structure [18]. Under different temperature conditions, the lap performance of modified bitumen waterproof membranes varies significantly: although low temperatures permit hot-melt construction, self-adhesive lap joints are generally not recommended for widespread application [19]. Construction techniques play a pivotal role in determining the performance of composite waterproofing layers. In this context, Chen et al. [20] evaluated the performance of polyurethane waterproof coatings in steel bridge deck waterproofing applications. Their findings indicated that polyurethane coatings exhibit superior tensile properties, water resistance, thermal stability, and bonding strength, thereby providing a theoretical basis for the design of effective waterproofing systems for steel bridge decks.

It should be noted that different waterproof materials exhibit significant differences in aging behavior and compatibility when used in composite applications. A systematic evaluation of composite performance, aging mechanisms, and construction adaptability of these materials is essential for developing high-performance and long-lasting waterproofing systems with enhanced aging resistance. Current research primarily focuses on the durability of commonly used SBS-modified bitumen, whereas studies addressing the compatibility among different waterproof membranes remain limited. To address this research gap, ultra-thin reinforced self-adhesive polymer-modified bitumen membranes, polymer self-adhesive membranes, and polymer-modified bitumen root penetration-resistant membranes were selected for compatibility testing. Composite lap and interface bonding tests were conducted to systematically assess membrane compatibility and evaluate key performance indicators, including tensile strength, peel strength, and low-temperature flexibility of the composite systems. Combined with scanning electron microscopy observations, the interfacial structural features and potential defects resulting from membrane compatibility were further elucidated. The findings provide a scientific basis for the composite application of multiple waterproof membranes in complex engineering environments and offer valuable guidance for optimizing waterproofing system design and enhancing long-term project durability.

## 2. Experimental

### 2.1. Materials

The experimental materials selected for this study were waterproof membranes manufactured by Beijing Oriental Yuhong (Beijing, China), including ARC polymer-modified bitumen root penetration-resistant waterproof membranes, FXZ-150 polymer self-adhesive waterproof membranes, and Shui Liton ultra-thin reinforced self-adhesive polymer-modified bitumen waterproof membranes. The key performance indicators of these materials are summarized in Table 1. The membrane specifications adopted in this study were selected based on mainstream engineering applications and were intentionally designed to evaluate the compatibility and potential synergistic effects of commercial bituminous layers with different thickness grades in composite waterproofing systems.

**Figure 2:**
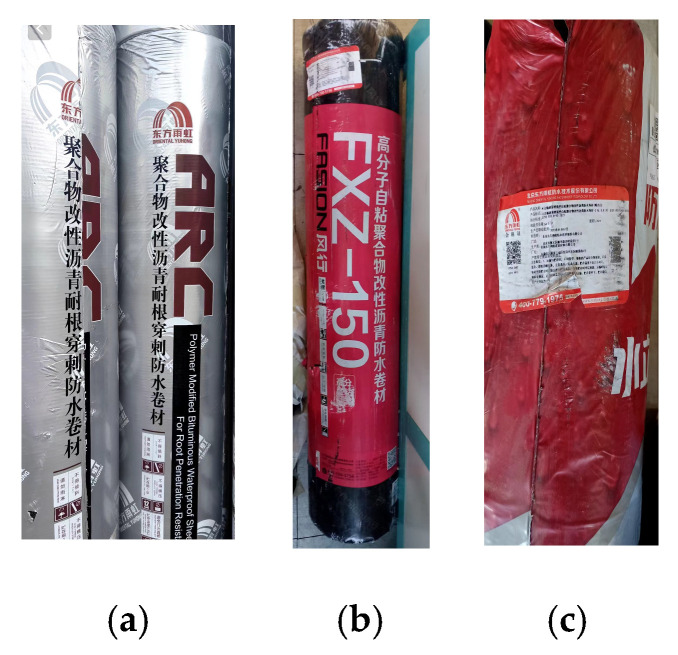
Waterproofing Membranes of Different Materials: (**a**) Polymer-modified Bitumen Root Penetration-resistant Waterproof Membrane; (**b**) Polymer Self-adhesive Waterproof Membrane; (**c**) Ultra-thin Reinforced Self-adhesive Polymer-modified Bitumen Waterproof Membrane.

A schematic diagram of the three types of Waterproofing Membranes is shown in Figure 2. The figure includes both the Chinese and English names of the products.

### 2.2. Pilot Program

(1) Specimen Preparation

During specimen preparation, the procedures strictly adhered to the requirements specified in Test Methods for Building Sheets for Waterproofing (GB/T 328.1-2007) [21]. To ensure sampling consistency, the membranes were uniformly cut along the width direction. To eliminate the potential influence of edge defects on test results, all specimens were taken from regions at least 150 mm away from the membrane edges. The long axis of each specimen was aligned with the longitudinal direction of the membrane so that the applied stress corresponded to the material’s actual service conditions. Specimen dimensions were selected according to the specific test requirements. For the low-temperature flexibility test, specimens with dimensions of 25 mm × 150 mm were used; for the tensile performance test, specimens measured 50 mm × 300 mm; and for the peel test in the compatibility evaluation, specimens measured 50 mm × 200 mm. All tests were conducted along the longitudinal direction to ensure consistency in mechanical loading conditions. It should be noted that reinforced waterproof membranes may exhibit a certain degree of material anisotropy due to the orientation of the reinforcement, which could influence fracture behavior. However, anisotropic effects were not explicitly investigated in this study, and the present analysis focuses on a comparative evaluation of mechanical performance under a uniform loading direction.

(2) Compatibility Test Procedures

**Figure 3:**
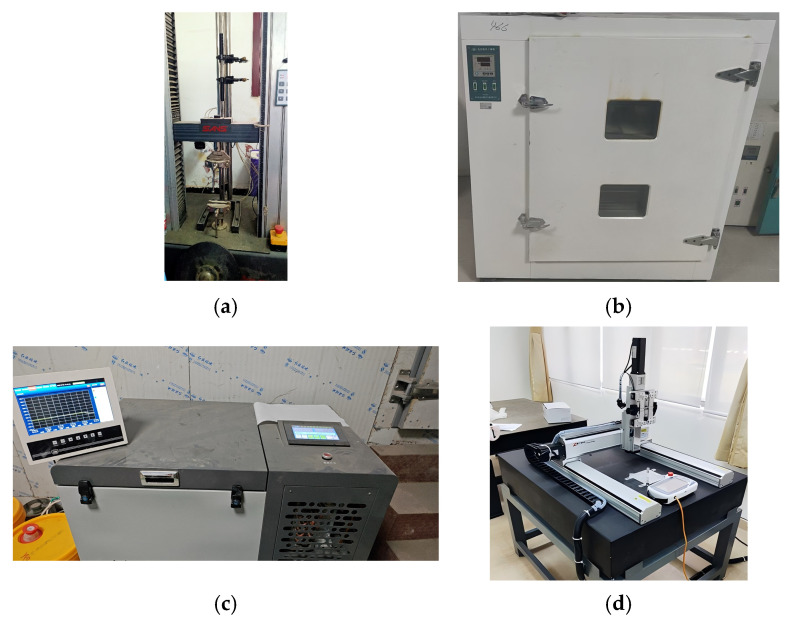
Test equipment: (**a**) Universal testing machines; (**b**) Thin film oven; (**c**) Low-temperature flexibility tester; (**d**) Test cutting machine.

This study evaluates the compatibility and performance evolution of three types of waterproof membranes under different aging conditions through systematic compatibility and performance tests. Initially, compatibility peel tests were conducted in the unaged state to determine the interfacial bonding strength between different membranes. Subsequently, the samples were subjected to thermal-oxidative aging for different durations (2, 5, 14, and 28 days), followed by tensile property and low-temperature flexibility tests. A comparative analysis was performed to assess changes in tensile strength, elongation at break, and low-temperature crack resistance at different aging durations. Thermal-oxidative aging was conducted using a Thin Film Oven (TFO) at a temperature of 80 °C for durations of 2, 5, 14, and 28 days [22]. After thermal-oxidative aging, the specimens were conditioned in a constant-temperature and constant-humidity incubator for 24 h prior to tensile and low-temperature flexibility testing. The relevant testing equipment is shown in Figure 3.

(3) Test Methods

The tensile performance tests were conducted in accordance with Test Methods for Building Waterproofing Membranes (GB/T 328.8-2007) [23]. A universal electronic testing machine was used to stretch specimens with dimensions of 200 mm × 50 mm at a constant crosshead speed of 100 mm/min, and tensile strength at break and elongation at break were recorded. The bonding performance and compatibility of the materials were primarily evaluated using seam peel tests. Seam peel performance tests were conducted in accordance with GB/T 328.8-2007 [23] and GB/T 328.20-2007 [24] using a universal electronic testing machine. Test specimens measured 200 mm × 50 mm, with a seam width of 100 mm, and the initial grip separation was set at 100 mm. During testing, the grips were displaced at a constant speed of 100 mm/min.

It should be noted that these viscoelastic membrane materials exhibit pronounced strain-rate sensitivity. Theoretically, an increased loading rate suppresses polymer chain relaxation, typically resulting in enhanced tensile strength and stiffness but reduced elongation. A loading rate of 100 mm/min was employed to ensure that the results are directly comparable with industrial standards and previously reported literature.

## 3. Results and Discussion

### 3.1. Tensile Properties

**Figure 4:**
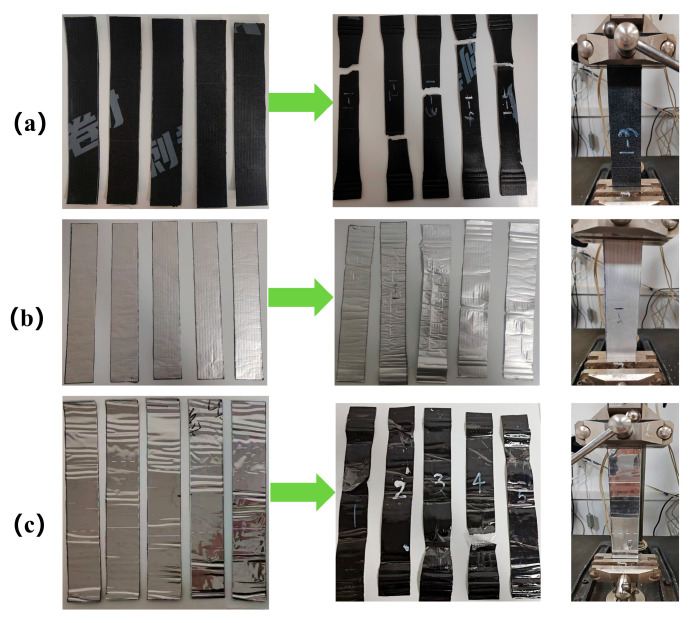
Tensile failure modes of unaged waterproof membranes: (**a**) root-resistant waterproof membrane; (**b**) polymer butyl-based waterproof membrane; (**c**) ultrathin reinforced self-adhesive bitumen waterproof membrane.

After thermal aging for 2, 5, 14, and 28 days, tensile tests were conducted on three types of membranes—ultra-thin reinforced self-adhesive polymer-modified bitumen membranes, polymer self-adhesive waterproof membranes, and root penetration-resistant polymer-modified bitumen membranes, as shown in Figure 4—and the results were compared with those obtained from unaged membranes.

(a) Unaged tensile specimens

Tensile tests conducted on three types of bitumen-based waterproof membranes revealed distinct differences in their mechanical behavior. The ultra-thin reinforced self-adhesive bitumen membrane exhibited superior ductility during tensile loading, withstanding substantial deformation and displaying pronounced plastic deformation at fracture, indicating strong adaptability under tensile stress. In contrast, the root penetration-resistant waterproof membrane exhibited a brittle failure mode, characterized by rapid crack propagation and localized fracture with minimal elongation.

From a mechanistic perspective, the incorporation of root-suppressive additives significantly alters the molecular dynamics of the bituminous matrix. These additives typically exist as dispersed phases or interact chemically with the polymer-modified network. They tend to restrict the segmental mobility of polymer chains and bitumen molecules through steric hindrance and interfacial interactions. This restriction reduces the free volume available for molecular rearrangement under tensile stress, thereby limiting the material’s capacity for plastic deformation. Consequently, the energy dissipation capacity of the material is significantly diminished. Furthermore, interfaces between additive particles and the viscoelastic matrix act as stress concentration sites. Under tensile loading, these localized stress peaks may exceed the cohesive strength of the binder, serving as nucleation sites for fracture initiation. This promotes rapid crack propagation, resulting in the characteristic brittle failure mode observed in root penetration-resistant membranes (RPRMs).

The polymer butyl-based waterproof membrane exhibited intermediate mechanical behavior, combining a certain degree of ductility with localized brittle fracture, suggesting moderate adaptability under tensile stress but comparatively lower tensile resistance.

**Figure 5:**
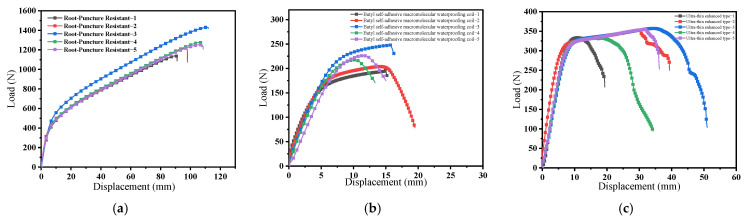
Tensile test results of different waterproof membranes: (**a**) root-resistant waterproof membrane; (**b**) polymer butyl-based waterproof membrane; (**c**) ultrathin reinforced self-adhesive bitumen waterproof membrane.

Figure 5 presents the tensile test results of three unaged waterproof membranes, with five replicates tested for each membrane type. The results demonstrate significant differences in tensile behavior among the three materials. The root penetration-resistant waterproof membrane (Figure 5a) exhibited a relatively high load-bearing capacity, reaching a maximum load of approximately 1400 N. However, with increasing displacement, the load increased gradually before abrupt fracture, indicating high tensile strength but limited ductility. The polymer butyl-based waterproof membrane (Figure 5b) exhibited a more stable load–displacement response, with a maximum load of approximately 250 N. The load increased uniformly with displacement, reflecting favorable tensile performance and good ductility, which enabled the membrane to sustain a certain degree of deformation prior to failure. The ultra-thin reinforced self-adhesive bitumen waterproof membrane (Figure 5c) displayed typical elastic–plastic deformation behavior during tensile loading, with tensile strength ranging from 300 to 360 N, which is consistent with the fracture patterns observed in Figure 4.

(b) Tensile specimens at different aging times

**Figure 6:**
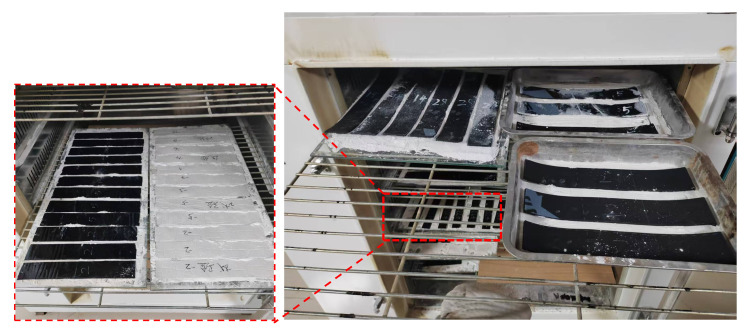
Thermal–oxidative aging process of different specimens.

**Figure 7:**
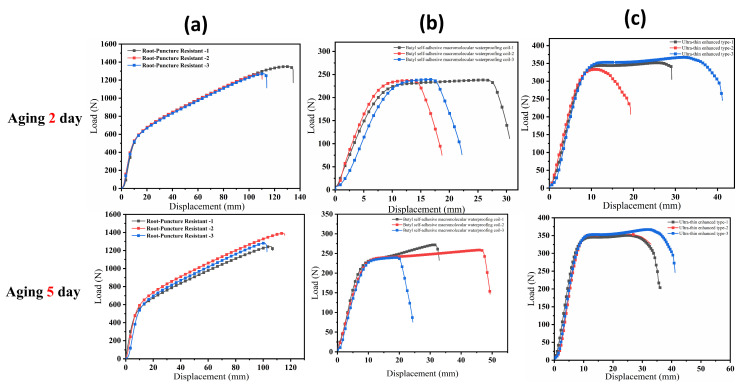

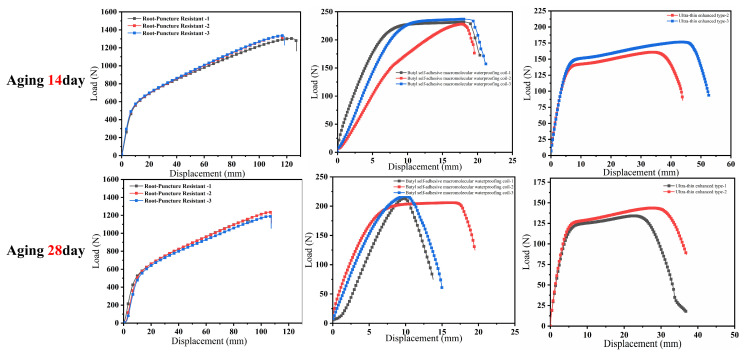
Tensile load–displacement curves of three waterproof membranes at different aging durations: (**a**) root-resistant waterproof membrane; (**b**) polymer butyl-based waterproof membrane; (**c**) ultrathin reinforced self-adhesive bitumen waterproof membrane.

The thermal–oxidative aging process in the aging chamber is illustrated in Figure 6, while the tensile test results under different aging durations are presented in Figure 7. For each condition, three replicate specimens were tested.

Figure 7 presents the tensile load–displacement curves of specimens subjected to different aging durations. From the evolution of the curves with aging time, it can be observed that the root penetration-resistant membrane initially exhibited high stiffness and peak load-bearing capacity; however, with prolonged aging, both the peak load and the corresponding displacement decreased rapidly, and the post-peak curve dropped steeply, indicating a typical brittle failure behavior. The polymer butyl membrane initially exhibited substantial deformability due to the flexibility of its molecular chains. Although its fracture load decreased only slightly with aging, its ductility was significantly reduced and its deformation capacity gradually diminished, ultimately exhibiting a transition from an initially compliant state to a brittle failure mode. The observed decline in ductility is primarily attributed to the volatilization of plasticizers and oxidative cross-linking reactions. These mechanisms reduce the free volume and restrict polymer chain mobility, thereby preventing effective chain uncoiling under tensile stress. This transition toward a brittle state highlights that resistance to oxidative hardening is critical for ensuring long-term mechanical stability.

In contrast, the self-adhesive bitumen membrane exhibited relatively stable curve patterns throughout the aging process, although its peak load showed the most pronounced decline over time. In this study, membrane ductility is quantitatively defined and reported as the elongation at break (%). This parameter represents the maximum strain the material can sustain prior to fracture and serves as the primary indicator of material flexibility and deformation capacity.

**Figure 8:**
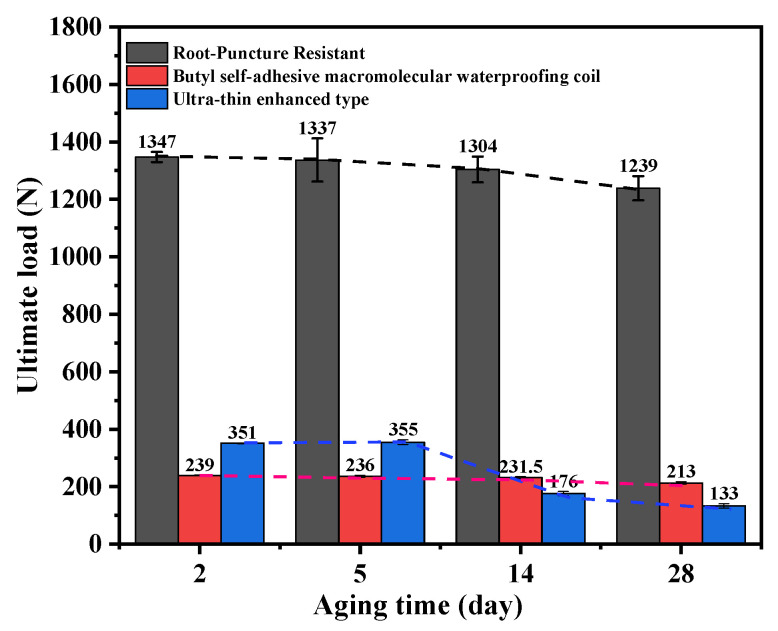
Variation curves of ultimate load for three waterproof membranes.

Each experimental condition was tested using three independent specimens, and the reported values represent the mean values with corresponding standard deviations. Figure 8 illustrates the variation in ultimate load among different waterproof membranes. To facilitate a direct comparison of aging resistance independent of absolute strength, the property retention rate, defined as the ratio of the aged value to the initial value, was calculated.

The root penetration-resistant membrane exhibited the highest initial ultimate load of 1347 N. Although the ultimate load decreased to 1239 N after aging, a high retention rate of 92.0% was maintained, indicating good durability and a slow degradation rate. The polymer butyl membrane exhibited a relatively low initial load-bearing capacity of 239 N; however, its reduction to 213 N was minor, corresponding to a retention rate of 89.1%. This result indicates that although its absolute strength is lower, the material exhibits high stability during aging. In contrast, the ultra-thin reinforced membrane exhibited an initial ultimate load of 351 N. Despite a slight short-term increase, the ultimate load declined sharply to 133 N, resulting in a markedly low retention rate of only 37.9%. This behavior reflects poor durability and severe long-term performance deterioration. Overall, the three membranes exhibited distinct aging patterns, which can be summarized as high strength with gradual degradation, low strength with relative stability, and moderate strength with rapid deterioration.

### 3.2. Low-Temperature Flexibility Analysis

The low-temperature flexibility tests of the self-adhesive membranes were conducted in accordance with the requirements of GB 23441-2009 [25], Self-adhesive Polymer-modified Bitumen Waterproof Membranes [18]. The crack temperatures obtained from the low-temperature flexibility tests for the ultrathin reinforced self-adhesive polymer-modified bitumen waterproof membrane and the polymer-modified bitumen root-resistant waterproof membrane, both in the unaged condition and after different levels of aging, are summarized in Table 2.

As shown in Table 2, the low-temperature flexibility of polymer-modified bitumen waterproof membranes deteriorated progressively with increasing aging duration. The root-resistant waterproof membrane exhibited superior low-temperature flexibility and aging resistance compared with the ultrathin self-adhesive bitumen membrane. After 28 days of aging, the cracking temperature of the root-resistant specimen increased only slightly from −35 °C to −28 °C (a 7 °C change), whereas the ultrathin self-adhesive specimen increased markedly from −28 °C to −18 °C (a 10 °C change). This enhanced performance of the root-resistant membrane can be attributed to the incorporation of specialized chemical root inhibitors, which confer greater structural stability. Mechanistically, Chemical Root Inhibitors (CRIs) act as thermally stable plasticizers that resist volatilization during aging. By maintaining free volume and providing steric hindrance against asphaltene agglomeration, they ensure the matrix retains sufficient stress relaxation capability and flexibility at low temperatures.

### 3.3. Compatibility Analysis

**Figure 9:**
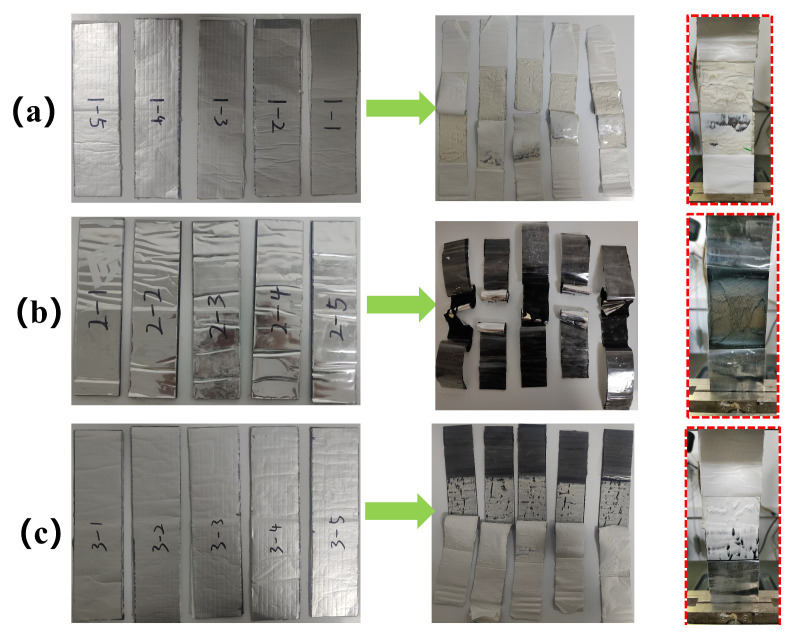
Overlap peel tests of different waterproof membranes: (**a**) polymer self-adhesive membrane with polymer self-adhesive membrane; (**b**) ultrathin reinforced self-adhesive bitumen membrane with ultrathin reinforced self-adhesive bitumen membrane; (**c**) polymer self-adhesive membrane with ultrathin reinforced self-adhesive bitumen membrane.

Compatibility between the ultrathin reinforced self-adhesive bitumen waterproof membrane and the polymer self-adhesive waterproof membrane was evaluated. The compatibility of each membrane with its corresponding material was examined through peel performance tests to assess their interfacial interactions and mechanical behavior. The experimental procedures followed the requirements of GB/T 328.20-2007 [24], and the loading process is illustrated in Figure 9. When two polymer self-adhesive membranes were overlapped and peeled, interfacial failure occurred due to the relatively thin adhesive layer. The adhesive was torn from the substrate surface, resulting in an irregular peel path. In contrast, the overlap peeling of two ultrathin reinforced self-adhesive bitumen membranes exhibited typical cohesive failure within the adhesive layer. The intermediate adhesive layer ruptured and partially remained on both substrate surfaces, with the dark self-adhesive bitumen undergoing internal fracture during peeling. For the overlap peeling between the ultrathin reinforced self-adhesive bitumen membrane and the polymer self-adhesive membrane, cohesive failure primarily occurred at the interface between the two adhesive layers, as shown in Figure 9c.

**Figure 10:**
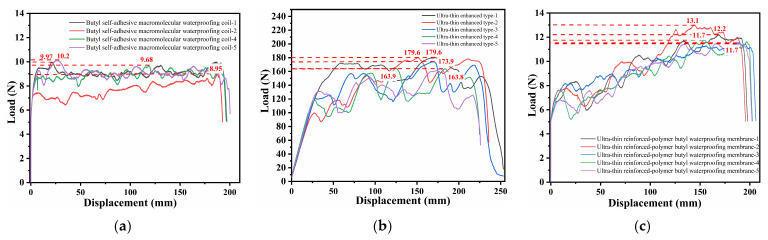
Peel strength tests of membrane compatibility: (**a**) polymer self-adhesive membrane with polymer self-adhesive membrane; (**b**) ultrathin reinforced self-adhesive bitumen membrane with ultrathin reinforced self-adhesive bitumen membrane; (**c**) polymer self-adhesive membrane with ultrathin reinforced self-adhesive bitumen membrane.

Figure 10 presents the peel test curves of three groups of materials. For the polymer self-adhesive membrane, the peel load remains stable at approximately 8–10 N, with smooth and relatively small fluctuations, indicating uniform interfacial bonding but limited load-bearing capacity. In contrast, the ultra-thin reinforced self-adhesive bitumen waterproofing membrane exhibits a substantially increased peel load of 120–200 N, characterized by pronounced peaks and fluctuations. The composite system of the two materials shows a maximum peel load stabilized at around 14 N, with curves displaying staged peaks and certain fluctuations. The reinforcement layer enhances load-bearing capacity while introducing saw-tooth fluctuations typical of staged failure. These peaks and drops correspond to fibril formation and rupture within the matrix, respectively. This progressive cohesive damage confirms that superior interfacial compatibility has shifted the weakest link from the interface to the bulk material, facilitating significant energy dissipation. Although variations among different samples are observed, the significant synergistic effect of material combination demonstrates good compatibility between the two systems.

## 4. Conclusions

Through systematic testing and comparison, this study investigates three types of bituminous waterproof membranes—namely the root-resistant membrane, the polymer self-adhesive membrane, and the ultra-thin reinforced membrane. Their mechanical properties, low-temperature flexibility, aging resistance, and compatibility were evaluated to clarify differences in load-bearing capacity, ductility, and long-term performance. The results reveal the respective advantages and limitations of these membranes in practical engineering applications, providing a basis for membrane selection and composite design. The main conclusions are summarized as follows:

(1) The three waterproof membranes exhibit distinct tensile characteristics: the root-resistant membrane demonstrates the highest load-bearing capacity but limited ductility; the polymer self-adhesive membrane shows relatively low load-bearing capacity but maintains stable performance; the ultra-thin reinforced membrane exhibits good initial ductility, yet its performance declines significantly with extended aging.
(2) Low-temperature flexibility testing shows that the root-resistant membrane undergoes the smallest variation in crack resistance temperature, indicating superior adaptability to low-temperature conditions and enhanced anti-aging performance. In contrast, the ultra-thin reinforced membrane exhibits rapid deterioration in low-temperature flexibility after aging.
(3) When the ultra-thin reinforced self-adhesive bituminous membrane is combined with the polymer self-adhesive membrane, the peel strength is significantly increased (14 N), with cohesive failure occurring predominantly within the adhesive layer. This result demonstrates a clear synergistic effect and confirms the strong compatibility between the two materials.

## Figures and Tables

**Table 1:** Characteristics of self-adhesive asphalt waterproofing membranes.

Membrane Type	Thickness (mm)	Low-Temperature Flexibility (°C)	Heat Resistance	Initial Elongation	Structural Feature	Polymer Matrix	Reinforcement
ARC-Polymer-modified Bitumen Root Penetration-resistant Waterproof Membrane	4.0	No cracking at −25 °C	No flowing or dripping at 105 °C for 2 h	Low	Thick modified bitumen layer	SBS-modified bitumen + Chemical Root Inhibitors	Pyramidal polyester felt
FXZ-150-Polymer Self-adhesive Waterproof Membrane	1.5	No cracking at −25 °C	No flowing or dripping at 80 °C for 2 h	High	Flexible polymer-based adhesive	Butyl Rubber (IIR) + Polyisobutylene	None
Shui Liton-Ultra-thin Reinforced Self-adhesive Polymer-modified Bitumen Waterproof Membrane	0.8	No cracking at −25 °C	No flowing or dripping at 70 °C for 2 h	Moderate	Reinforced, thin adhesive layer	SBS-modified bitumen	Glass fiber

**Table 2:** Cracking temperature for low-temperature flexibility test.

Aging Time (Day)		0	2	5	14	28
Cracking temperature (°C)	Root-Puncture Resistant waterproofing coil	−35	−33	−32	−32	−28
	Butyl self-adhesive macromolecular waterproofing coil	−28	−25	−24	−22	−18

## Data Availability

The original contributions presented in this study are included in the article. Further inquiries can be directed to the corresponding authors.
